# The accuracy and safety of CT-guided iodine-125 seed implantation assisted by 3D non-coplanar template for retroperitoneal recurrent carcinoma

**DOI:** 10.1186/s12957-020-02087-0

**Published:** 2020-11-25

**Authors:** Weijuan Jiang, Ping Jiang, Shuhua Wei, Yuliang Jiang, Zhe Ji, Haitao Sun, Jinghong Fan, Weiyan Li, Yuxia Shao, Junjie Wang

**Affiliations:** Department of Radiation Oncology, Peking University Third Hospital, No.49 Huayuan North Road, Haidian District, Beijing, 100191 People’s Republic of China

**Keywords:** LDR brachytherapy, Seed implantation, 3D-printing non-coplanar template, Retroperitoneal carcinoma, Accuracy

## Abstract

**Purpose:**

To investigate the accuracy, dosimetric parameters, and safety of 3D-printing non-coplanar template (3D-PNCT)-assisted CT guidance for radioactive iodine-125 (125I) seed implantation brachytherapy (RSI-BT) for retroperitoneal recurrent carcinomas

**Methods and materials:**

We enrolled 15 patients with 17 retroperitoneal recurrent carcinomas after external beam radiotherapy (EBRT). All patients received CT-guided 125I RSI-BT assisted by 3D-PNCT successfully. We compared the original needle insertion position, angular, and the needle tip distance deviations of preoperative plan with that of intraoperative in brachytherapy treatment planning system (B-TPS). The dosimetric parameters of RSI-BT were evaluated on preoperative plan, intraoperative real-time plan, and postoperative plan, including D90, D100 (the dose to 90% and 100% of the target volume), V100, V150, and V200 (the volume receives 100%, 150%, and 200% of the prescribed doses). The quality assurance of RSI-BT evaluated on conformal index (CI), external index (EI), and homogeneity index (HI) of the targets were compared among preoperative plan, intraoperative real-time plan, and postoperative plan. The perioperation complications and RSI-BT-related toxicity were assessed.

**Results:**

The median follow-up was 8.2 months (range 1–18.5 months). One patient was lost to follow-up after RSI-BT. Fourteen patients were assessed for response rate and toxicity. The mean entrance point distance deviation for all 165 needles was 4.50 ± 4.10 mm (range, 0–30). The mean angular deviation was 2.70 ± 3.00° (range, 0–20). The needle tip distance deviation was 6.90 ± 6.00 mm (range, − 30–28). D90 for preoperative plan, intraoperative plan, and postoperative plan were 140.55 ± 23.93, 124.25 ± 28.04, and 128.98 ± 22.75, respectively. There was significant difference between D90 of preoperative plan with that of intraoperative plan (*p* = 0.036). Four lesions reached CR, six lesions reached PR, three lesions were SD, and three lesions were PD. Four patients with moderate pain became mild, and two with mild pain relieved completely after RSI-BT. The other parameters showed no differences among preoperative plan, intraoperative plan, and postoperative plan. The perioperative complications were observed in four patients, including three patients of grade 1 and one patient of grade 2. No ≥ grade 3 side effects were observed.

**Conclusion:**

CT-guided 125I RSI-BT assisted by 3D-PNCT was a safe, accurate, and feasible strategy for recurrent carcinomas located in the retroperitoneal regions.

## Introduction

The anatomic construction in retroperitoneal region was deep and complex due to a majority of important organs located there, such as the blood vessels and spinal cord. The most common carcinomas in retroperitoneal regions were metastasis originated from cervical cancer, pancreatic cancer, gastric cancer, and adrenal metastasis, while the primary carcinoma was mainly soft tissue sarcoma [1–5]. The standard treatment approach was EBRT plus chemotherapy for recurrent carcinoma after surgery or metastasis in the retroperitoneal region. However, the majority of recurrent patients after EBRT or EBRT combined with chemotherapy were often resistant to re-radiation or chemotherapy [6–10]. The salvage approach for recurrent patients was always deemed as a challenge, most of which was palliative treatment and mainly for relieve of the symptoms. The response rate (RR) of chemotherapy, which is the second line or salvage treatment regimen for cervical, pancreatic, and gastric carcinoma, was unfavorable [11–13]. Most patients suffered from severe pain and suboptimal quality of life (QOL).

125I RSI-BT belongs to low-dose rate brachytherapy (LDR-BT) which delivers locally high doses inside the targets and rapidly drops off surrounding the normal tissues [14]. Transperineally ultrasound (US)-guided 125I RSI-BT was a standard modality for prostate cancer because of the excellent outcomes and minimally invasive procedure compared with surgery or EBRT [15, 16]. The disadvantages of US-guided RSI-BT included (1) two-dimensional image reconstruction and low resolution; (2) unsuitable for carcinomas located in the head and neck, thoracic, retroperitoneal, pelvic, and spinal cord due to air or bony construction interferences; (3) the available commercial brachytherapy treatment planning system (B-TPS) was special for prostate carcinoma, with all needle arrangement kept in parallel and high dependence on ultrasound guidance; (4) the available template was rigid and only for parallel needle trajectory, which was unsuitable for non-coplanar insertion. The suboptimal dose conformity for special location tumors was unavoidable. With the development of 3D-printing techniques, the individualized template was invented and named as 3D-printing non-coplanar template (3D-PNCT) [17]. However, 3D-PNCT combined with CT guidance for long distance puncture pathway achieved optimal dosimetry conformality in the targets. Thus, the accuracy and safety of 3D-PNCT-assisted CT guidance RSI-BT were investigated in this study.

## Methods and materials

### Patient indication selection

Totally, 15 patients with 17 lesions from recurrent carcinomas located in retroperitoneal regions who received EBRT were enrolled in this study from August 2016 to September 2019 (Table 1). The pre-RT doses ranged from 30 to 80 Gy. The median tumor volume was 23.1 cc (range, 5.1–68.6 cc). The indications for selected patients in this study were as follows: (1) pathological or radiological diagnosis was confirmed; (2) the diameter ≤ 5 cm and no invasion to intestinal tube or spine; (3) preoperative plan showed that the needle channel pathway and the prescribed doses were satisfactory; (4) predicted survival time ≥ 3 months. The exclusion criteria were as follows: (1) severe coagulation functional disorder, (2) tumor invasion into the spine or intestine, (3) and preoperative plan showed that there was no satisfied needle puncture pathway. The study was approved by the ethics committee of our hospital.

**Table 1:** General information

	Number	Percentage (%)
Gender		
Male	7	46.7
Female	8	53.3
Age, years		
Median (range)	58 (38–78)	
Primary tumor location		
Esophagus	3	20
Pancreas	1	6.7
Cervix	4	26.7
Corpus uteri	3	20
Liver	1	6.7
Colon	1	6.7
Ureter	1	6.7
Cardia	1	6.7
Pathological type		
Squamous cell carcinoma	7	46.7
Adenocarcinoma	2	13.3
Hepatocellular carcinoma	1	6.7
Urothelium carcinoma	1	6.7
Endometrioid adenocarcinoma	3	20
Neuroendocrine carcinoma	1	6.7
Previous treatment		
EBRT	15	
Prescribed doses (median, range)	50.4 (30–80)	
Implantation location		
Retroperitoneal lymph node	13	76.5
Adrenal gland	3	17.6
Pancreas	1	5.9

### Patient preparation and preoperative plan

Patients were set-up on the CT simulator and immobilized at prone position with vacuum mattress. Both native and contrast CT scan were performed with 5-mm thickness before RSI-BT. The CT scan slices were transferred into B-TPS (Beijing University of Aeronautics and Astronautics and Beijing Astro Technology Co., Ltd) for preoperative plan. Planning system source data originated from the latest official manuscripts of the American Association of Physicists in Medicine (AAPM) [18, 19].

### The principle of preoperative plan design

**Fig. 1:**
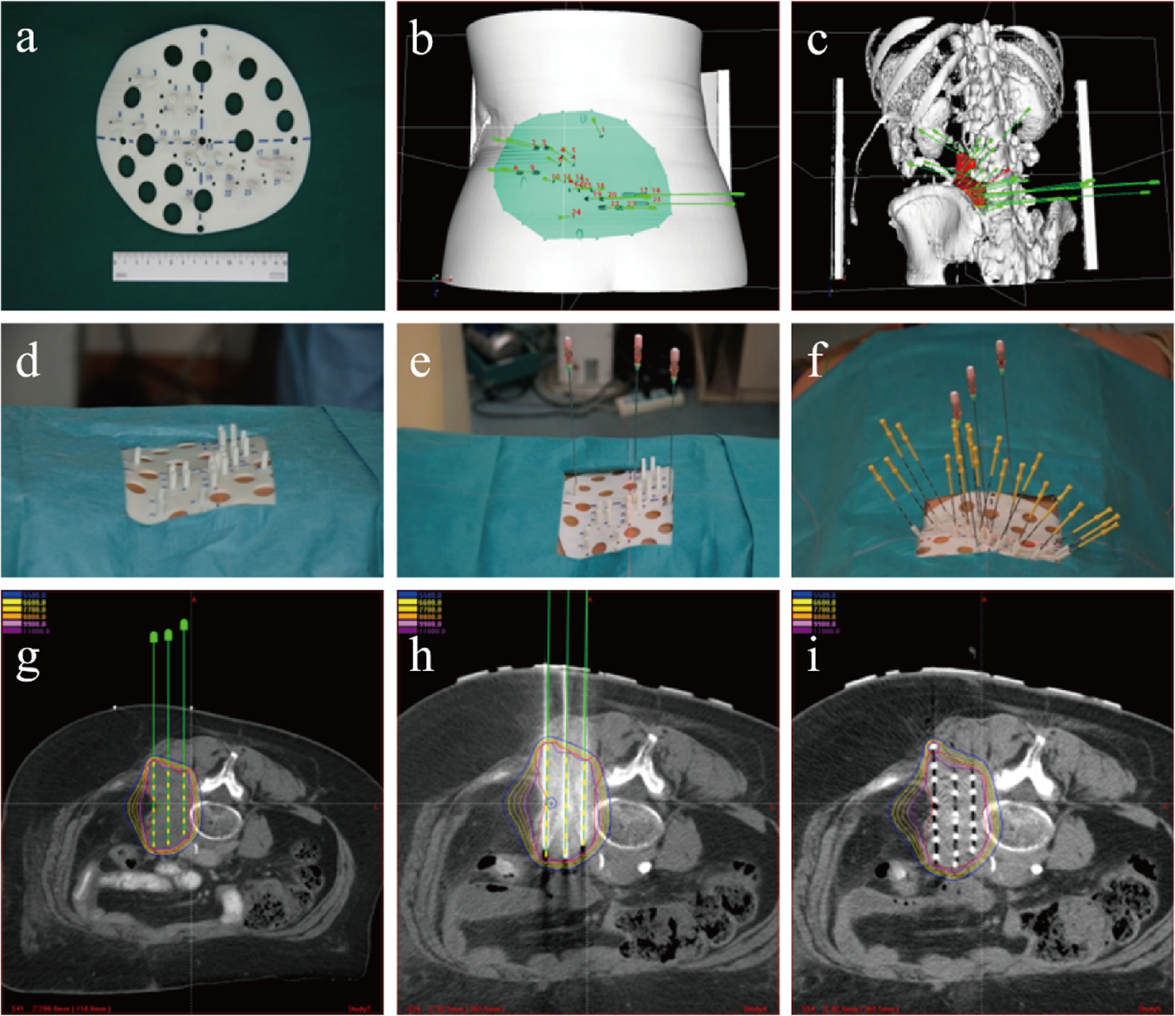
The work-flow of 3D-PNCT-assisted CT guidance RSI-BT. **a** 3D-PNCT. **b** 3D-PNCT reconstruction on CT images. **c** 3D model view of bony anatomy and the puncture needle distribution reconstruction. **d** 3D-PNCT set-up: **e** stable needle insertion based on preplan, **f** seed needle insertion, **g** preplan, **h** intraoperative real-time plan, and **i** postoperative plan

The needles should be kept in parallel, with distance of 1–1.5 cm. If the organs at risk (OARs) are interference for the needles, non-coplanar needle distribution was adapted to satisfy the target dose conformity and the lowest doses to normal tissue. Then, we delineated targets and OARs and defined prescribed doses and limitation of OARs. Clinical target volume (CTV) was expanded from gross tumor volume (GTV) by 5–6 mm in three dimensions. Prescribed doses were 110–160 Gy; the radioactivity of 125I seed was 0.4–0.7 mCi (the seed model was 6711-1985, Shanghai GMS Pharmaceutical Co., Ltd). The principles of seed distribution were sparse in the center and dense at the peripheral zone of the targets (Fig. 1).

### 3D-PNCT design and production

The B-TPS data were imported into 3D imaging and reverse engineering software for individualized digital modeling design. 3D-PNCT was obtained by a 3D curing rapid prototyping machine and the material processing of medical curing resins. The 3D-PNCT contained certain information such as body surface characteristics, X- and Y-axis coordinates, 2–3 stable needles, and dummy needle channel holes.

**Fig. 2:**
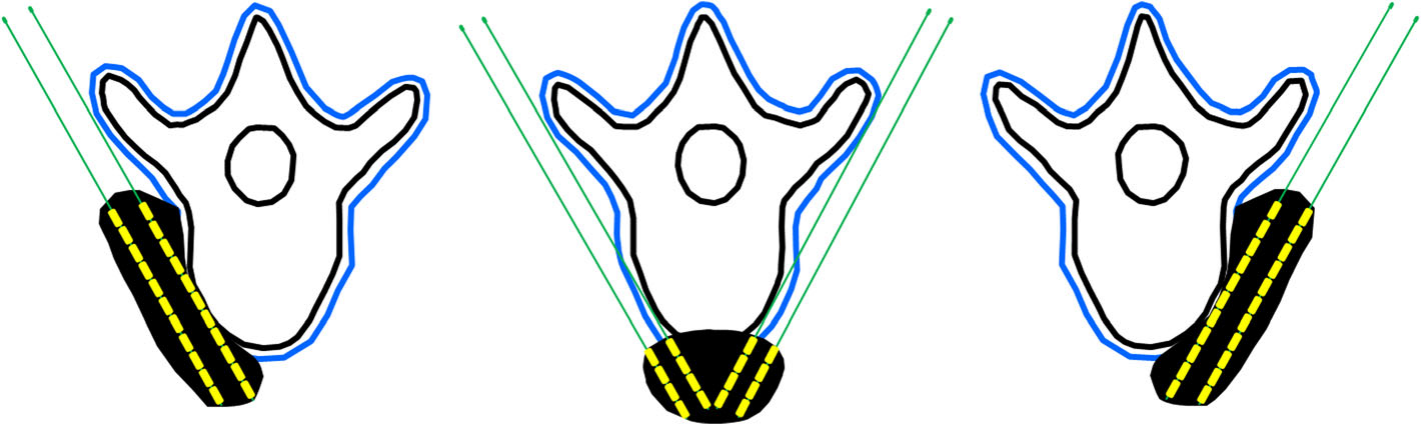
The pattern of recurrent retroperitoneal carcinoma

We classified recurrent retroperitoneal carcinoma into 3 subgroups according to the recurrent locations referenced to the spinal cord. The definition of subgroup included the following: (1) type 1, the recurrent tumors located in the left side of the spinal cord and the front margin did not spread to the middle line, the needles are inserted from the left side; (2) type 2, the recurrent tumor is located before the front edges of the spinal cord, the needles are inserted from two sides; (3) type 3, the recurrent tumors located in the right side of the spinal cord and the front margin did not spread to the middle line of the spinal cord, the needles are inserted from the right side (Fig. 2).

### The work-flow of 125I RSI-BT

The procedures of 125I RSI-BT were as follows: (1) patients were set-up again with template fixed on the patient body by 2–3 stable needles; (2) CT-scan was performed to verify the stable needles position. If the deflection errors were > 2 mm, we adjusted the needles’ position until the deviation ≤ 2 mm. (3) The other implant needles were inserted into the targets; (4) CT scan was performed again to check the tips of the needles’ position. If the deflection errors were > 2 mm, we made a fine adjustment until the errors were ≤ 2 mm; (4) 125I seeds were implanted with applicators according to the preoperative plan and the needles withdraw to the skin below 1 cm; (5) CT scans were conducted again to verify the seed distribution. If the seed distribution did not meet the requirements of preoperative plan, seed savage implantation was performed immediately, and all the needles were taken out; (6) CT scan was performed again and transferred the slices into the B-TPS for postoperative dose parameter calculation [17]. After RSI-BT, the patients returned to patient wards and received perioperative prophylactic antibiotics and hemostasis for 1 day and discharged 24 h later.

### Postoperative plan for dosimetric verification

The D90, D100, V100, V150, and V200 were recognized as the dosimetric parameters of tumor target. The quality assurance comparison of RSI-BT involved CI, EI, and HI among preoperative plan, real-time plan, and postoperative plan.

### Definition of end points

#### Main end points

(1) To evaluate the accuracy of needle distributions, we compared the preoperative plan with intraoperative on B-TPS. The CT images in the preoperative plan were fused with intraoperative real-time CT scan images depending on bone construction as the references. The needle tip positions, the needle angles, and the tip depth were measured. (2) The D90 (doses delivered to 90% of the target volume), D100, V100 (the percentage of the target volume receiving at least 100% of the prescribed doses), V150, and V200 were calculated. (3) The quality of RSI-BT in the preoperative, real-time, and postoperative plan targets were compared with CI, HI, and EI.

#### Secondary main point

(1) Perioperation side effect assessment included bleeding, fever, infection, fistula; (2) the radiation-related complications were assessed according to RTOG Common Toxicity Criteria; and (3) pain relief rate was evaluated.

### Follow-up

After RSI-BT, routine follow-up was performed every 3 months in the first 2 years and every 6 months from 3 to 5 years, followed by annual evaluation. CT scans of the thorax and abdomen were part of the follow-up for contrast. Patients underwent clinical evaluation and laboratory testing. The evaluation of efficacy was based on the Response Evaluation Criteria in Solid Tumors (RECIST) v1.1, including complete response (CR), partial response (PR), progressive disease (PD), and stable disease (SD). Adverse reactions were evaluated by the Common Terminology Criteria for Adverse Events (CTCAE) v 4.0 (CTCAE 2010) [20, 21]. Pain was assessed using a numerical rating scale (NRS) which was categorized into five grades: 0 for no pain, 1–3 for mild pain, 4–6 for moderate pain, 7–9 for severe pain, and 10 for unbearable pain. The pain score in 1 month after the treatment was compared with that of pre-operation.

### Statistical analysis

The characteristics of patients were expressed as continuous variables and/or categorical variables. Continuous variables were compared using the *t* test or rank-sum test, whereas the categorical variables were compared using the chi-square or Fisher’s exact test. ORR was expressed based on the number and percentage of patients. The SPSS 21.0 software (SPSS, Chicago, IL) was used for statistical analysis. The *P* value < 0.05 was considered as statistical significance.

## Results

The median follow-up time was 8.2 months (range, 1–18.5 months). One patient was lost to follow-up. The 15 patients with 17 lesions were enrolled for accurate assessment, and 14 patients were involved for side effect analysis. The median GTV in the preoperative plan, intraoperative plan, and postoperative plan were 26.41 ± 16.2, 26.71 ± 17.43, and 26.72 ± 17.46, respectively (*p* > 0.5). The mean needle tip deviation for all 165 needles was 4.50 ± 4.10 mm (range, 0–30, *p* < 0.001). The mean angular deviation was 2.70 ± 3.00° (range, 0–20, *p* < 0.001). The needle depth deviation was 6.90 ± 6.00 mm (range, − 30–28, *p* < 0.001) (Table 2). D90, D100, V100, V150, and V200 for preoperative plan, intra-operative plan, and postoperative plan were 140.55 ± 23.93, 124.25 ± 28.04, 128.98 ± 22.75; 66.70 ± 16.77, 58.14 ± 21.24, 61.51 ± 15.86; 24.83 ± 15.94, 22.71 ± 12.10, 23.64 ± 13.75; 20.02 ± 13.32, 17.45 ± 10.15, 18.74 ± 11.53; and 14.51 ± 11.02, 12.38 ± 8.07, 23.59 ± 8.97, respectively. The CI, EI, and HI were 0.54 ± 0.16, 0.50 ± 0.15, 0.50 ± 0.17; 0.90 ± 1.00, 0.91 ± 0.90, 1.06 ± 0.99; and 0.22 ± 0.07, 0.25 ± 0.09, 0.21 ± 0.08, respectively (Table 3). There were no statistically significant differences in D100, V100, V150, V200, CI, EI, and HI in GTV between preplan and intraoperative plan, preoperative plan and intraoperative plan, and intraoperative plan with that of postoperative plan (*p* > 0.05), while there was only significant differences between D90 (*p* < 0.05) of preoperative plan with that of intraoperative plan. There were no differences between intraoperative dose optimization with that of postoperative plan.

**Table 2:** The deviation of position, angle, and distance of needles between preoperative plan and intraoperation plan in B-TPS

No. of needles	Deviation of position (mm)			Deviation of angle (°)			Deviation of distance (mm)		
	Mean	Range	SD	Mean	Range	SD	Mean	Range	SD
165	4.5 (0.000)	0–30	4.1	2.7 (0.000)	0–20	3.0	6.9 (0.000)	− 30~28.1	6.0

**Table 3:** The dosimetric parameters of 125I seeds implantation (*x* ± *s*)

	Preoperative	Intraoperative	Postoperative	P*	P**	P***
Number of needles	10.76 ± 4.05	9.76 ± 3.80	9.82 ± 3.75	0.010	0.007	0.332
Number of seeds	48.00 ± 16.80	46.82 ± 18.25	47.88 ± 18.75	0.595	0.962	0.070
GTV volume (cm3)	26.41 ± 16.2	26.71 ± 17.43	26.72 ± 17.46	0.585	0.629	0.952
D90	140.55 ± 23.93	124.25 ± 28.04	128.98 ± 22.75	0.036	0.102	0.338
D100	66.70 ± 16.77	58.14 ± 21.24	61.51 ± 15.86	0.169	0.459	0.500
V100	24.83 ± 15.94	22.71 ± 12.1	23.64 ± 13.75	0.142	0.317	0.129
V150	20.02 ± 13.32	17.45 ± 10.15	18.74 ± 11.53	0.058	0.302	0.056
V200	14.51 ± 11.02	12.38 ± 8.07	23.59 ± 8.97	0.119	0.451	0.072
CI	0.54 ± 0.16	0.50 ± 0.15	0.50 ± 0.17	0.68	0.342	0.985
EI	0.90 ± 1.00	0.91 ± 0.90	1.06 ± 0.99	0.981	0.553	0.119
HI	0.22 ± 0.07	0.25 ± 0.09	0.21 ± 0.08	0.380	0.637	0.117

*Refers to preoperative vs. intraoperative

**Refers to preoperative vs. postoperative

***Refers to intraoperative vs. postoperative

When it comes to response rate at 3 months after seed implantation, CR was observed in four lesions. PR was shown in six lesions. SD was observed in three lesions. PD was observed in three lesions. Among them, four patients with moderate pain became mild; two with mild pain were relived completely after RSI-BT. One patient had pain again 4 months later, and the other five patients had pain control until the last follow-up or death. The median time to achieve pain control was 8 months (range, 4–13 months). The perioperation complications were observed in four patients, including three of grade 1 and one of grade 2. No ≥ grade 3 side effects were observed (Table 4).

**Table 4:** Perioperative and postoperative complication of seeds implantation

		RTOG/CTC scoring schema			
		G1	G2	G3	G4
Perioperative	Pneumothorax	0	1	0	0
	Hematemesis	1	0	0	0
	Hemorrhage of digestive tract	1	0	0	0
	Fever	0	0	0	0
	Infection	0	0	0	0
Postoperative	Skin	1	0	0	0

## Discussion

High-dose-rate BT (HDR-BT) is a very important modality in RT, and commonly used in breast cancer, cervical cancer, prostate cancer, and skin cancer treatment [22, 23]. The outcome of HDR-BT combined with surgery for selected recurrent soft tissue carcinomas were favorable, however, with the complication rates ranged from 15 to 50% [24]. The 125I RSI-BT belongs to LDR-BT and has advantages as follows: (1) single performance; (2) real-time image guidance; (3) the local boost dose to target; (4) minimal invasion and fast recovery.

In order to expand the indications of RSI-BT, CT guidance technique was integrated into RSI-BT in 2002 in China, which was used to treat head and neck, thoracic, abdomen, and spinal vertebrate carcinoma [25–30]. The CT-guided 125I RSI-BT was a very safe and effective approach for anti-cancer treatment, especially for recurrent or metastatic carcinomas. The majority of recurrent carcinomas located in retroperitoneal regions had lost the opportunity to further resection or re-radiation after EBRT due to the surrounding OARs’ dose limitation such as the intestine, spinal cord, and blood vessels. Yao et al. reported that 17 patients with 19 retroperitoneal lymph node recurrences after EBRT underwent CT-guided 125I RSI-BT. The actuarial D90 of postoperative plan was 100–198 Gy (median, 126.5 Gy). Nine patients with pain decreased to mild pain 1–3 weeks after RSI-BT. Pain-free survival time ranged 2–15 months (median, 5 months). The overall survival (OS) rate was 100%. The median local control (LC) time was 15 months. The 1- and 2-year LC rate was 63.2% and 42.1%, respectively. Twelve patients (70.6%) died of distant metastasis. Two patients (11.8%) survived with distant metastases and without evidence of local recurrence. Median OS time was 10 months. The 1- and 2-year OS rate were 38.1% and 15.3%, respectively. No major complications related to RSI-BT occurred during or after treatment [31]. There were some drawbacks as follows: (1) It is a long period to train a skilled puncture personnel; (2) the quality control of RSI-BT was not often concomitant with preoperative plan design due to OAR interferences such as blood vessels, nerve, and bone construction. The analysis of CT-based dosimetry revealed that dose coverage of RSI-BT postoperative plan was often lower than that in preoperative plans; (3) the operation time average was 2–3 h because CT scan was conducted to check every needle position for several times. Thus, investigators explored the possibility of template-assisted CT guidance to overcome these limitations, which theorized the precision placement of seeds to the periphery of the targets in a highly conformal manner, ensured good tumor control, and minimized the doses to the surrounding OAR.

With the development of 3D-printing techniques, the 3D-printing substance was explored across the medical field. Gross et al. used stereolithography printers to print the temporal bone anatomy of patients with congenital aural atresia to plan atresia plasticity [32]. D’Urso et al. published their early study with 3D-printed models of craniofacial and maxillofacial defects in 45 patients [33]. Their experience showed improved measurement accuracy and suggested modest improvement in operative time while improving patient education [34]. BT plays an important role in cancer treatment as monotherapy or combined with EBRT. The key techniques were the image guidance combined with template assistance which assured the applicators or needles placed into the targets accurately as preoperative plan designed, especially for the breast, prostate, and skin cancer [14, 15]. Those cancer anatomic locations were relatively simple, and the applicators or needles can easily be inserted into the interested targets and kept in parallel arrangements. The optimal dose patterns of the targets were achieved to maximize the doses to the cancer and minimize the doses to normal tissues. However, the carcinomas located in the head and neck, thoracic, retroperitoneal, pelvic, and spinal cord sites were very different from the above location. The rigid template for prostate or cervical cancer cannot satisfy the requirements for complex anatomy construction and shape of tumors with RSI-BT.

Huang et al. first reported the accuracy of RSI-BT in 31 patients with recurrent and locally advanced head and neck malignant tumors. The preliminary study confirmed that this approach was easier and more accurate. The D90, V100, and V150 all meet the treatment requirements [35]. The carcinomas that occurred in the head and neck regions were relatively stable and superficial, so it is relatively easy for template to fix with the patient anatomic construction, and the needle puncture deviation was infrequent. We applied the individualized template for the carcinomas in a deep location and long-distance puncture pathway. The body 3D-printing individual, digital, and coordinate template was invented in 2015. The 3D-PT was classified into 3D-printing coplanar template (3D-PCT) and 3D-PNCT. The indications of 3D-PCT were only for needle insertion kept in parallel way, and the needle hole interval was 0.5–10 mm which assured that the target dose pattern meets the preoperative plan requirements. When the needle pathway was impeded by OARs with 3D-PCT assistance, the needles were adjusted to the near holes to keep away from OARs or established an artificial channel with puncher for bone construction. The indications of 3D-PNCT can apply to almost all the carcinomas in different locations for RSI-BT; the target dose conformality of which was optimal [36]. The advantages of 3D-PNCT included the following: (1) The X- and Y-axis coordinate located in the center of the template which can match with the marked lines on the patient body surface; (2) extra 2–3 stable needle holes designed on the template was used to immobilize the template on patient body. The stable needle position should be designed near the bony construction as references and easy to compare with preoperative plan images; (3) the dummy needle channels were designed to overcome the organ motion during operation. Salvage seed implantation can be performed immediately due to suboptimal dose pattern on the real-time plan optimization, in order to avoid the patients returning to hospital for second time seed implantation. The increasing accuracy and universality of RSI-BT promoted it to be an alternative minimally invasive and precise ablation approach which was deemed as stereotactic ablation brachytherapy (SABT).

The effective treatment modality for recurrent carcinomas in retroperitoneal locations still faces a giant challenge, especially for recurrence after EBRT. Our preliminary study showed the possibility and safety of 3D-PNCT-assisted CT-guided RSI-BT, which suggested that intra-operative D90 was significantly different with that in preoperative plan, and there was no significant difference between preoperative plan and postoperative plan through real-time dose optimization. The needles’ position deviation for recurrent carcinomas located in retroperitoneal region might occur, due to the long distances puncture, the organ motion, and OAR interferences. The difference of needles’ tip position, angles, and depth among preoperative plan, intraoperative plan, and postoperative plan were all significant, but no significant differences were seen on the CI, EI, and HI among preoperative plan, real-time plan, postoperative plan, which suggested that the intraoperative needle position adjustment and dose optimization were very important. The postoperative plan D90 of RSI-BT would reach the preoperative plan designed requirements even for carcinomas located in depth position. The perioperation complications were moderate. The radiation-related toxicity rates were acceptable.

## Conclusion

3D-PNCT-assisted CT-guided RSI-BT is a safe and effective approach in treatment of recurrent carcinomas in retroperitoneal regions. It is worth warranted to conduct more multiple, prospective, and randomized clinical trials to compare the long-term efficacy of 125I RSI-BT with second line treatment.

## Data Availability

No additional data are available.
